# A Simulation Curriculum for Ground and Air ECMO Transport

**DOI:** 10.15766/mep_2374-8265.11508

**Published:** 2025-03-18

**Authors:** Daniel Shouldice, August Felix, Kyle Danielson, Matthew Plourde, Nicholas J. Johnson, Andrew Latimer, Richard Utarnachitt, Jenelle Badulak

**Affiliations:** 1 Attending Physician, Department of Emergency Medicine, University of Washington School of Medicine; 2 Critical Care Fellow, Department of Anesthesiology and Pain Medicine, University of Washington School of Medicine; Flight Physician, Airlift Northwest; 3 Flight Nurse and Director of Operations, Airlift Northwest and UW Medicine; 4 Flight Nurse and Education Manager, Airlift Northwest and UW Medicine; 5 Associate Professor, Division of Pulmonary Critical Care and Sleep Medicine, Department of Emergency Medicine, University of Washington School of Medicine; 6 Associate Professor, Department of Emergency Medicine, University of Washington School of Medicine; Flight Physician, Airlift Northwest and UW Medicine; 7 Assistant Professor, Division of Pulmonary Critical Care and Sleep Medicine and Division of Cardiothoracic Surgery, Department of Emergency Medicine, University of Washington School of Medicine; † Co-primary author

**Keywords:** ECMO Transport, Extracorporeal Membrane Oxygenation, Simulation, Resuscitation, Cardiac Arrest, Shock, Communication Skills, Interdisciplinary Medicine, Critical Care Medicine, Emergency Medicine, Surgery - Cardiothoracic, Clinical/Procedural Skills Training

## Abstract

**Introduction:**

The use of extracorporeal membrane oxygenation (ECMO) for cardiopulmonary failure is increasing. ECMO transport teams are needed to expand patient access. Our goal was to design and implement a simulation curriculum for an ECMO transport team.

**Methods:**

Using Kern's model, we developed a curriculum, built an ECMO protocol, and performed a pilot simulation. We conducted a needs assessment survey to evaluate presimulation confidence performing ECMO transport procedures using a 5-point Likert scale (1 = *strongly disagree*, 5 = *strongly agree*). We used the results to refine a simulation script encompassing transport logistics, ambulance and aircraft loading/unloading, and emergencies. A simulation mannequin with attached ECMO circuit and all necessary equipment was used to simulate transfer of patients receiving ECMO and to ensure learners could manage in-flight emergencies. After implementation, we distributed a postsimulation survey to assess changes in confidence.

**Results:**

The needs assessment was implemented for four physicians, five ECMO specialists, and 11 flight nurses. The needs assessment revealed 95% of respondents were ECMO transport novices. Mean confidence scores for transporting an ECMO patient were low (3.4). The finalized simulation was implemented for 10 members, eight of which completed the needs assessment and postsimulation surveys. Confidence scores improved overall (3.0 to 4.3) and for emergency procedures: air entrainment (3.0 to 4.6), pump failure (3.3 to 4.6), patient loading/unloading (2.8 to 4.1), and use of transport protocols/checklists (3.5 to 4.8).

**Discussion:**

We successfully developed a simulation-based ECMO transport curriculum, which resulted in enhanced confidence in multiple aspects of ECMO transport.

## Educational Objectives

By the end of this activity, learners will be able to:

1. Demonstrate the ability to safely transport a patient on extracorporeal membrane oxygenation (ECMO).
2. Discuss the equipment, steps, and team members involved in transport.
3. Manage specific ECMO emergency scenarios (air entrainment and pump failure) during transport.
4. Exhibit closed-loop communication during the transport process.

## Introduction

Extracorporeal membrane oxygenation (ECMO) is used to rescue patients with cardiopulmonary failure. Use of ECMO is increasing dramatically,^1^ but it is a complex technology that requires specialized equipment, skills, and knowledge. Because of its complexity, it is only available in specialized centers, thus the need for an ECMO transport team.^2^

The ECMO system is a mechanical device that can be used for temporary partial or complete cardiopulmonary bypass for refractory cardiopulmonary failure once other conventional therapies have been exhausted. ECMO has two main configurations: venoarterial (VA ECMO) and venovenous (VV ECMO). The first, VA ECMO, drains blood from the vena cava using a cannula placed in a central vein and subsequently pumps blood outside of the body at a rate of 3–5 L/min through an artificial lung. This provides oxygenation to the blood as well as removal of carbon dioxide. The blood is pumped back into the arterial system via a cannula placed in a central artery. As this system provides both gas exchange and hemodynamic support, it can be used for cardiac and pulmonary support. VV ECMO drains blood using a similar intravenous catheter; however, with VV ECMO, the blood is returned to the venous system, through a central vein. This allows for only pulmonary support.^3^ Some of the more common indications for initiation of ECMO include severe acute respiratory distress syndrome, severe asthma exacerbation, massive pulmonary embolism, cardiotoxic ingestion, cardiac arrest, and cardiogenic shock. ECMO serves as a bridge to native organ recovery, transplantation, or durable device placement.

Because of the complex, multidisciplinary care required for patients receiving ECMO, specialized teams are required to safely place patients on ECMO and subsequently transport them to ECMO centers. The Extracorporeal Life Support Organization (ELSO), which publishes standards for ECMO care, recommends the development and training of specialized ECMO transport teams utilizing protocols and checklists.^4^

ECMO transport is logistically difficult and combines challenges in the physical transfer of not only the patient, but also the ECMO cannulas and pump, ventilator, intravenous lines, and multiple medication infusions. The management of patients receiving ECMO uses a team-based approach that includes physicians, critical care nurses, and ECMO specialists or perfusionists. Failure to adequately account for these many variables during transport or movement of ECMO patients can have significant consequences. There have been several studies regarding the feasibility of ECMO transport,^5,6^ and some that highlight a standardized protocol or approach.^7,8^

Simulation has been used throughout medical education to both teach and reinforce principles in management of many different patient scenarios and is particularly useful for high stakes clinical interventions.^9,10^ ECMO simulation has been shown to be a viable method of improving knowledge and confidence in trainees, and is particularly useful to prepare team members for ECMO emergencies.^11,12^ There is preexisting literature on ECMO training^13^; however, there does not appear to be established literature for development of an ECMO transport team curriculum.

We describe the development and implementation of a simulation-based, interprofessional curriculum to build an ECMO transport team.

## Methods

### Development

This simulation was developed at the University of Washington (UW Medicine) as part of the ECMO transport curriculum. The UW ECMO transport protocol (Appendix A) was built using examples from peer institutions, ELSO guidelines,^4^ and existing literature.^8,14–18^ The simulation script (Appendix B) was created to guide the learners through execution of the transport protocol and select ECMO emergency scenarios (air entrainment, pump failure).

Using Kern's model for curriculum development,^19^ a targeted needs assessment survey (Appendix C) was developed to best identify background demographics and prior experience of the group as well as to ascertain which elements of ECMO transport the group felt most uncomfortable with so that they could be simulated to increase confidence.

Initially a pilot simulation was developed to demonstrate feasibility of the project. The simulation script and transport protocol were initially trialed with four ECMO transport team members. Feedback was used to improve the simulation and transport protocols. During this test run, the team loaded into and out of a fixed wing aircraft and traveled to and from a hospital in Seattle via ambulance to identify potential high-risk failures and integrate these scenarios into the finalized simulation script. Subsequent iterations of the simulation were run in a more simplified environment limited to one physical location (simulation center).

Target learners included members of the ECMO transport team. Preparatory education for each team member was completed prior to this simulation:

• ECMO physician: completion of the UW ECMO Provider Course, which includes a 4-hour patient management workshop and 8 hours of online didactic material, independent management >20 ECMO patients, and board certification in intensive care or cardiothoracic surgery.
• ECMO specialist: completion of the UW Specialist Course, which includes 30 hours of didactic and simulation education as well as ongoing supervised bedside training. Further selection of flight team members was conducted after an audition to demonstrate rapid response to simulated circuit emergencies.
• Perfusionist: completion of accredited 2- to 4-year perfusion program.
• Flight nurse: completion of the 2-hour UW Bedside ICU Nurse ECMO Course.

### Equipment/Environment

Several different physical locations were used throughout the duration of the simulation, including the following:

1. Intensive care unit hospital room (or simulation room)
2. Ambulance
3. Medical transport aircraft

Recommended equipment for the simulation included the following:

• ICU bed
• Flight transport gurney
• Low-fidelity mannequin
• Transport ventilator with tubing attached to the mannequin
• ECMO pump and water-filled practice circuit attached to the mannequin
• Portable monitor with tablet to display simulated vital signs (telemetry, arterial line monitoring, pulse oximetry), and clinical monitoring device cables attached to the mannequin
• Infusion pumps (x8) with associated tubing connected to the mannequin
• Ambulance and/or medical aircraft
• Optional: Impella or intra-aortic balloon pump console with tubing attached to the mannequin
• Video recording device (optional)
• ECMO and flight equipment bags (Appendix A)
• ECMO transport protocol (Appendix A)
• Simulated patient data sheet (Appendix B)

### Personnel

In the simulation, the team of learners was composed of a diverse group of clinicians, which included a physician, two critical care flight nurses, and either an ECMO specialist or perfusionist. This was based on both team design from previous literature as well as feasibility given physical constraints of ground and air transport vehicles. Each provider was given certain responsibilities based on their role on the team (Appendix A, pages 1 and 2).

The simulation facilitator was a critical care physician who was able to provide background information about the simulated patient including history of present illness, hospital course, and current physical examination, as well as current ventilator and ECMO pump settings (Appendix B). The facilitator was also in control of changing vital signs and manipulating the ECMO circuit throughout the simulation depending on the clinical scenario. Optionally, an EMS crew may be utilized as well to provide transportation throughout the necessary portions of the simulation.

### Implementation

This simulation was implemented over multiple locations. The introductory phase of the simulation occurred in a generic conference room (to simulate the ECMO hospital and aircraft hangar) where the physician and ECMO specialist or perfusionist were introduced and provided with a copy of the ECMO transport protocol and asked to review team member roles, activation process, patient data sheet, equipment checklist, vehicle seating assignments, and the checklist provided to the referring hospital (Appendix A, pages 1–8). The facilitator introduced the simulation goals to the team members and subsequently provided pertinent history, hospital course, physical exam, current medications/infusions, and ventilator and ECMO pump settings to the team (Appendix B). They were allowed to ask any related or relevant questions at that time. Team members were then instructed to start the transport protocol (Appendix A, pages 9 and 10) by gathering supplies, packing bags, and performing equipment checklists.

The next phase occurred in a simulated referring hospital ICU room (Appendix D, location 1). The ECMO transport team members then received hand off from the referring providers, evaluated the patient, performed ECMO circuit change or cannulation, changed over medications and other devices, completed time out for patient transfer onto a stretcher, and performed the predeparture equipment checklist (Appendix A, pages 11–14).

An ambulance (Appendix D, location 2) was then utilized for the next portion of the simulation. The patient was wheeled from the simulated ICU, loaded into the ambulance using the time out for patient transfer, and then the checklists were completed to ensure all equipment was properly connected inside the vehicle (Appendix A, page 15). At this time, the facilitator generated vital sign changes and caused several ECMO emergencies to be managed by the team while in transit (Appendix B, Appendix D scenario A and B). The facilitator rode in the front passenger seat of the ambulance, with access to air entrainment tubing and ability to modify vital signs on the monitor, and the four-person ECMO team traveled in the patient compartment with the mannequin. To increase fidelity, circuit emergencies were simulated while the ambulance was in motion.

The patient was then transported to the fixed wing aircraft (Appendix D, location 3), loaded and unloaded, and then loaded back into and out of the ambulance using the same checklists as above (Appendix A, page 15). Finally, the patient was wheeled from the ambulance back to the simulated hospital room where the team transferred the patient to the hospital bed using the time out for patient transfer. A handoff to the receiving hospital providers (facilitator) was completed, and posttransport tasks were completed including the transport debrief form (Appendix A, pages 16 and 17). The simulation in its entirety took approximately 2 hours. See Appendix B for full simulation and critical actions.

The simulation was performed twice with no repeat participants. Given the limitations of individual schedules and logistics of coordinating with ground and air transport vehicles, only a limited number of team members were able to complete initial simulation curriculum after taking the pretest assessment.

### Debriefing

Standard simulation debriefing was performed by asking the team to recap what occurred during the simulation, what went well and what they would like to change next time (see below). Subsequently, a debriefing form, which is also part of the ECMO transport protocol, was completed. After the debriefing, all learners were given a postsimulation assessment similar to the presimulation assessment they had completed prior to the simulation (Appendix C).

Questions related to the overall simulation included the following:

1. Is there a volunteer who would briefly summarize what occurred during the simulation?
2. What do you feel went well overall?
3. What could be done differently?
4. How did you feel the communication went during the simulation?
5. What parts of the simulation were the most difficult?
6. Do you have any suggestions for improvement of the transport protocol?

Questions related to the critical actions included the following:

1. How did you identify air entrainment or pump failure during transport?
2. How do you manage air entrainment during transport?
3. How do you manage pump failure during transport?
4. What were the most important initial steps after the ECMO circuit emergencies were identified?

### Assessment

The facilitator reviewed the critical actions checklist and, with gentle prompting, ensured each step was completed by the learners prior to moving to the next phase of the simulation. The learners received feedback during their debriefing session immediately after the simulation. Feedback from the learners was used to improve the simulation and transport protocols. Evaluation of the transport curriculum also included a postsimulation assessment that evaluated the target learners’ confidence in specific aspects of the aircraft transport and transport emergencies.

## Results

The targeted needs assessment (pretest) was completed by 20 team members (69% response rate), of which four were physicians, five were ECMO specialists or perfusionists, and 11 were critical care transport nurses. Of the respondents, 95% had previously performed less than five ECMO patient transports. Confidence in various aspects regarding ECMO transport was assessed using a 5-point Likert scale (1 = *strongly disagree*, 5 = *strongly agree*). In our evaluation of the learner responses, we interpreted scores of 1–3 as lower confidence, as our goal was to have learners report final confidence scores higher than either low confidence (scores of 1 or 2) or neutral (score of 3).

In the pretest, mean confidence for the overall transport of ECMO patients among the team members was 3.4 on a 5-point Likert scale (Figure). Confidence in various aspects of aircraft transport and specific ECMO emergencies improved between pretest and posttest (Figure).

**Figure.**
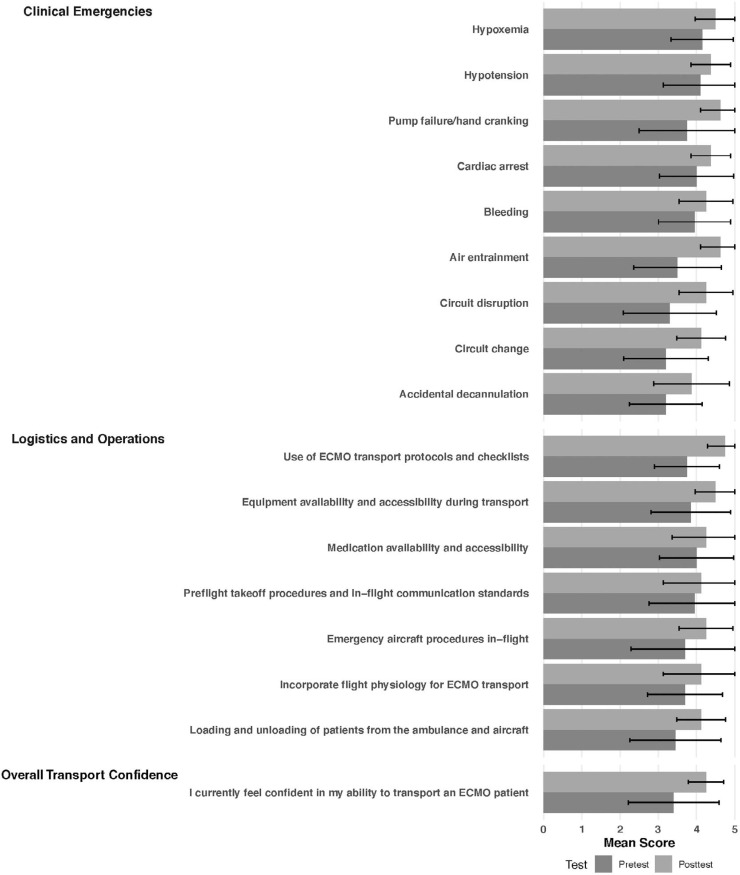
Mean confidence scores rated on a 5-point Likert scale (1 = *strongly disagree*, 5 = *strongly agree*) for clinical emergencies, logistics and operations, and overall transport capability before (pretest) and after (posttest) simulation training (bars indicate standard deviation). Abbreviation: ECMO, extracorporeal membrane oxygenation.

Ten of the team members who completed the pretest went through the simulation curriculum, eight of whom completed the postsimulation assessment (posttest). As referenced in the methodology section, the initial simulation curriculum was performed twice with no repeat participants. Each session was composed of at least one physician, two critical care flight nurses, and either an ECMO specialist or perfusionist. Given scheduling limitations and the availability of ground and air transport vehicles, only a limited number of team members were able to complete initial simulation curriculum after taking the pretest assessment. The posttest was sent within 24 hours after the simulation and aimed to assess changes in attitude and confidence in various aspects of ECMO patient transport.

The mean overall confidence score improved after the simulation to 4.3 (Figure). Confidence in many areas that were surveyed increased following the institution of the simulation curriculum (Figure), including the loading and unloading of patients using the transport protocols (2.8 to 4.1), use of the transport protocols and checklists (3.5 to 4.8), and confidence in specific ECMO emergencies such as air entrainment (3.0 to 4.6) and pump failure (3.3 to 4.6).

All participants demonstrated the ability to communicate appropriately among team members, and accurately follow the steps outlined in the ECMO transport checklist. During the debrief and feedback from the posttest, learners reported that the simulation was valuable, especially with the repeated physical transport experience of the mannequin and real medical equipment.

Since implementation of this curriculum, we have performed 41 successful ECMO transports without any significant clinical complications. The curriculum and transport protocols are continuously modified and improved using the debrief form completed at the end of each simulation and real patient transport mission.

## Discussion

We built a successful transport curriculum to train a diverse group of clinicians, which include critical care flight nurses, bedside specialists, perfusionists, and critical care physicians, to safely transport patients on ECMO. The curriculum primarily focused on the physical and operations aspects of ECMO patient transport with emphasis on standardized protocols. The secondary focus was on the clinical management of ECMO circuit emergencies that may arise during transport.

Many previous studies have highlighted the feasibility of ECMO transport as well as the safety, especially when team members are knowledgeable about the process and follow a standardized course.^5–8,14–18^ Given the lack of preexisting literature regarding the development of a specific curriculum to teach these team members, our simulation curriculum fills a current void in the literature.

The simulation curriculum itself is a viable method of teaching, training, and increasing the confidence of qualified health care providers for the safe transportation of patients on ECMO. We were able to show in multiple domains, including overall confidence and specific areas related to transport and ECMO emergencies, that this simulation curriculum was able to increase confidence levels in clinicians with diverse background training. The team subsequently brought these skills to real-life scenarios, successfully executing numerous ECMO transports without complication. We used feedback that was obtained from the needs assessment (pretest) in order to identify scenarios that the learners felt would be the most applicable and helpful for their training. Feedback obtained from the debriefing sessions that are held after each simulation is also used on a continual basis to continue to improve the simulation curriculum and ECMO transport protocol.

Our overall intent is to provide interested programs with a blueprint to conduct high-fidelity ECMO transport simulation. One limitation of this simulation curriculum is the high usage of resources. We understand that many interested programs may not have all the recommended resources to perform the simulation exactly as described. For example, it may be difficult to coordinate the use of an ICU room, ambulance, or aircraft all for use during a simulation. We felt that a core goal was the mastery of safe patient transfers and familiarity with our equipment, thus we made it a priority to dedicate these resources to this curriculum. For programs without these assets, this simulation could effectively be performed with lower resource utilization. Some of the most important aspects of the simulation are good team communication, following a standardized checklist, and mastering the skill of safe patient transfers. This can effectively be rehearsed without an ambulance or aircraft; however, it is still important to obtain a portable ECMO pump and have an accompanying low-fidelity mannequin. If hemodynamic and respiratory monitors are not available, the facilitator can also verbally relay changes in patient status to the team as needed. This curriculum can also be modular and can be broken down into specific portions that may be more pertinent to certain programs.

Finally, another limitation of our study is its primary outcome being a measurement of learner confidence rather than a more objective outcome. In the future we plan to continue implementing the simulation curriculum and hope to assess knowledge retention and clinical metrics related to ECMO transport safety at multiple time points in the future.

We require all UW ECMO transport team members to complete this training prior to patient transport, which has enabled us to swiftly build a confident ECMO transport team that has completed multiple safe missions. This curriculum can be used for other institutions with ECMO programs who need to build a transport team.

## Appendices


ECMO Transport Protocol.docxECMO Transport Logistics and Emergency Simulations.docxECMO Transport Needs Assessment.docxECMO Simulation Images.docx
*All appendices are peer reviewed as integral parts of the Original Publication.*
