# Supplementary material for: A Simulation Curriculum for Ground and Air ECMO Transport
Source: MedEdPORTAL. 2025 Mar 18;21:11508. doi: 10.15766/mep_2374-8265.11508 (PMC11913753; doi:10.15766/mep_2374-8265.11508)
Supplement: Supplementary file 1 — ECMO Transport Protocol.docxECMO Transport Logistics and Emergency Simulations.docxECMO Transport Needs Assessment.docxECMO Simulation Images.docx [file mep_2374-8265.11508-s001.zip › A. ECMO Transport Protocol.docx]

Appendix A: UW Medicine ECMO Retrieval Team Protocol

Description: This protocol is the comprehensive framework for all key components of ECMO transport. It includes team composition and roles, background training, and available modes of transport. It also includes flow diagrams for team activation and how to arrange personnel and equipment. Additionally it includes checklists for: patient data collection, equipment and medications, a debrief script, and checklists for required tasks at each timepoint from pre-departure to post-transport. It should be used as a reference by ECMO teams to develop and refine their ECMO transport protocol. The images included in this appendix are author owned.

Retrieval Team Members:

1. Primary transport: If the patient is *not* currently on ECMO:
   1. ECMO physician x2 (cannulation team)
   2. ECMO specialist/perfusionist
   3. Flight RN x2
2. Secondary transport: If the patient *is* currently on ECMO:
   1. ECMO physician (capable of performing circuit change)
   2. ECMO specialist/perfusionist
   3. Flight RN x2

Description of Team Member Roles:

1. ECMO Physician
   1. Patient management decisions
   2. (Performs cannulation/circuit change)
   3. Leads checklists and time-outs
2. Specialist/perfusionist
   1. Responsible for UW ECMO bags, circuit
   2. Manages ECMO circuit
   3. Manages Hemachron
   4. Manages Impella, IABP
3. Flight RN
   1. Responsible for flight bags, ventilator, monitor, IV pumps, iSTAT, blood
   2. Manage medications, infusions, blood product administration
   3. Manage ventilator
   4. Lead patient movement logistics (load/unload/transfer)

ECMO Retrieval Team Training Requirements

1. Flight RN
   1. Initial:
      1. ECMO didactic and simulation training session
      2. Transport simulation (hospital, ground ambulance, aircraft), familiar with protocol and checklists
   2. Maintenance:
      1. Twice yearly simulation training or patient transport runs
2. Physician, specialist/perfusion
   1. Medical flight company paperwork completion (emergency contact, passport)
   2. Aircraft flight safety training (can do just-in-time training)
   3. Flight physiology lecture (recording available)
   4. Transport simulation (hospital, ground ambulance, aircraft), familiar with protocol and checklists
   5. (physician cannulation/circuit change: out of state license & emergency credentialing at referring hospital)

Mode of Transportation:

Transfer Center will discuss mode of transportation with medical flight company including weather or aircraft related considerations

1. Ground:
   1. Ambulance requirements:
      1. Type I or Type III Ambulance (squared/boxed)
      2. Functional inverter
      3. Multiple oxygen hook-ups (2 or more)
      4. dedicated rigs suitable for Critical Care / ECMO transportation
   2. Ambulance crew must be made aware of size and number of staff and equipment to be transferred.
2. Fixed Wing: Pilatus PC-12 or Learjet
   1. Maximum of 5 care team members (not including pilot) can travel via fixed wing with a patient on board
   2. Learjet: not enough room for intra-aortic balloon pump + ECMO

**ECMO Transport Team Activation Tree**

**
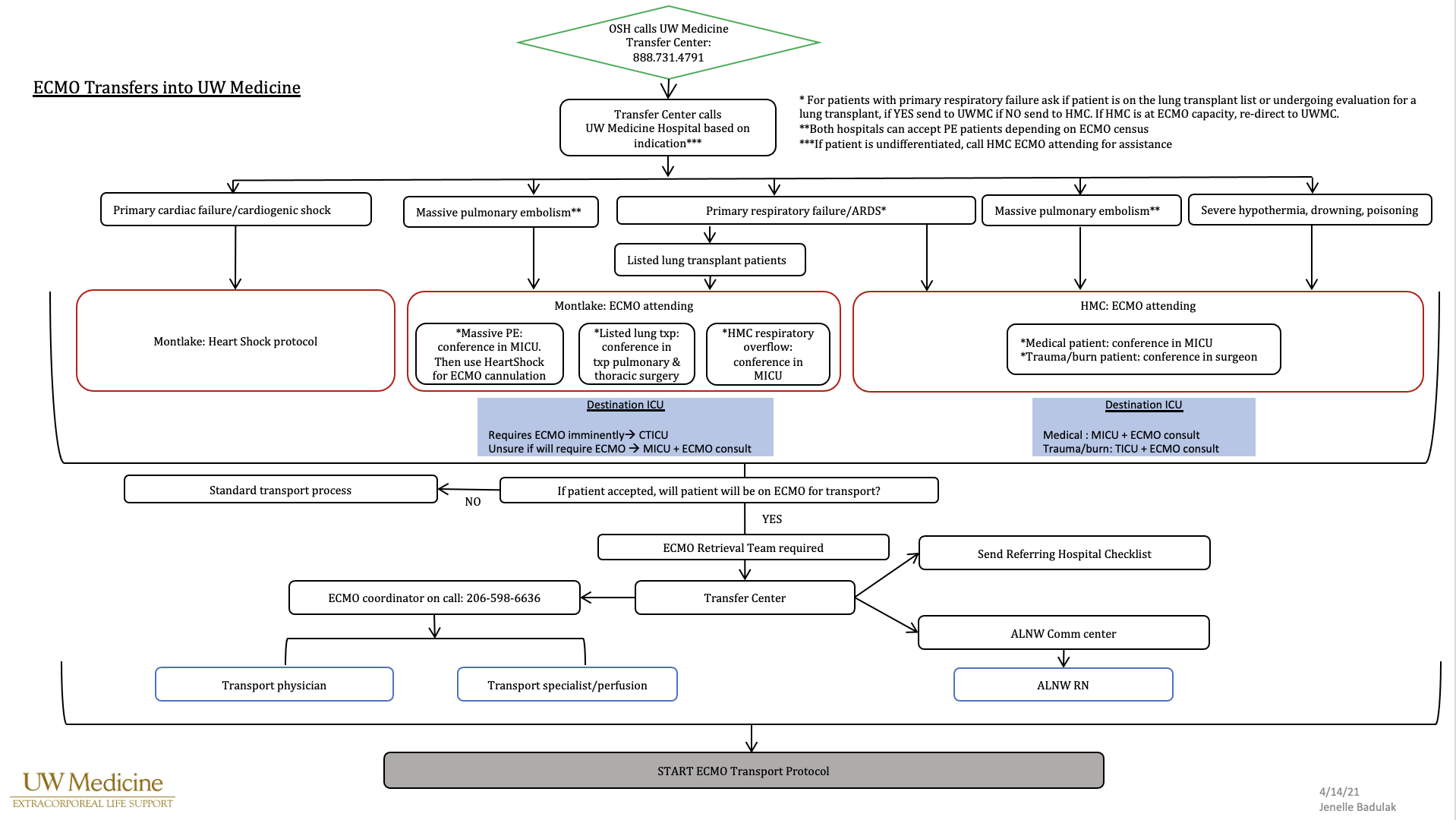
**

**ECMO Patient Data Sheet**

Recommended data to obtain prior to departure from ECMO Hospital:

1. Patient name, birth date & age
2. Height & weight
3. Referring hospital
4. Name and phone number of referring physician
5. General diagnosis and past medical history
6. Indication for ECMO
7. Last ABG: pH____ PaCO_2_:____ PaO_2_:____ HCO_3_:____ SaO_2_%: ____ Lactate: ____
8. Last BMP, CBC, LFTs
9. Last coags (PTT, INR, ACT)
10. Last vital signs:
    1. Heart Rate
    2. Blood Pressure
    3. SpO_2_
    4. Pulmonary artery catheter (PA pressures, CI, SvO2)
11. Ventilator settings:
    1. Rate
    2. Tidal volume or inspiratory pressure
    3. FiO_2_%
    4. PEEP
12. Vasoactive / inotropic medications
13. Sedation
14. Recent neuro exam
15. Average hourly urine output, dialysis?
16. Current IV access (central and peripheral)
17. IVC filter?
18. Results of echocardiogram: R or L ventricular dysfunction? Valvulopathy?
19. Impella or IABP? Settings?
20. If on ECMO:
    1. Type of system (cardiohelp, centrimag, rotaflow, tandem life, etc)
    2. Run start time/date
    3. Blood flow & RPM
    4. Sweep gas flow rate
    5. Circuit pressures
    6. Cannulation Configuration: VA, VV, VVA, size of cannulas, distal perfusion?
    7. LV vent?
    8. Anticoagulation
    9. Any circuit problems?

**ECMO Equipment Checklist**

**
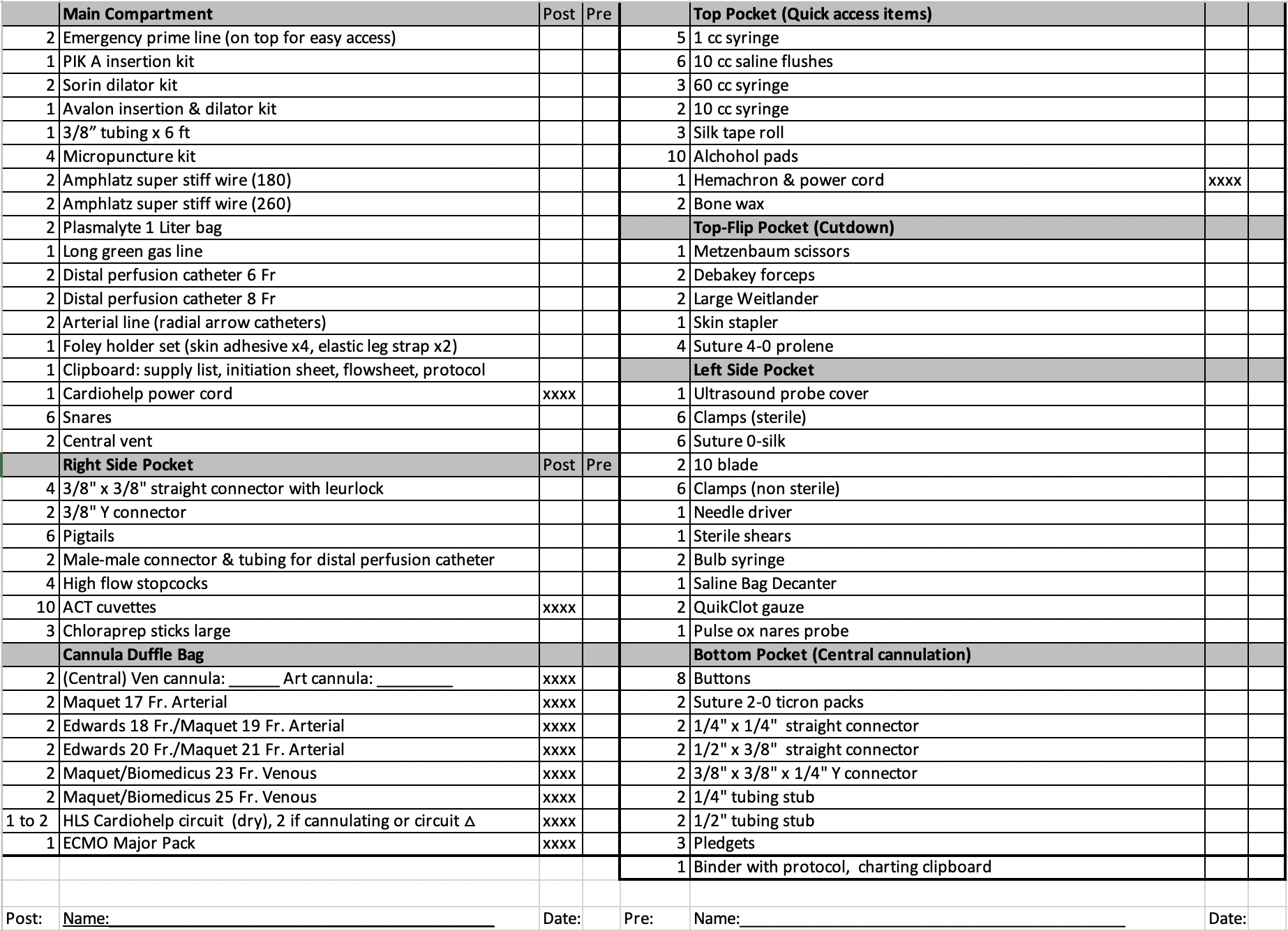
**

ECMO Major Pack includes: basin, laparotomy drape, sterile gloves (sizes 6.5-8), 4x4” gauze, sterile gowns, hair covers, surgical masks, face-shields, 6-pack of sterile blue towels

**ECMO Medication List**

Medical flight company primary bag

| ⮚  10cc, 3cc, 1cc, and 10cc NS FLUSH x 3 EACH  ⮚  NS 250cc x 1 |
| --- |
| ⮚  ADENOSINE 6mg x 3 |
| ⮚  AMIODARONE 150mg x 3 |
| ⮚  ASA X 1 BOTTLE  ⮚  ATROPINE x 2 (BRISTOJECT) |
| ⮚  BENADRYL 50MG x 1 |
| ⮚ CA CL x 1 (BRISTOJECT) |
| ⮚  D50 x 1 |
| ⮚  EPI 1:1000 1ML AMP x 1  ⮚  EPI x 3 (BRISTOJECT) |
| ⮚  ETOMIDATE 20mg X 2  ⮚  LIDOCAINE x 1 (BRISTOJECT) |
| ⮚  MAG SULFATE 5GM x 1 |
| ⮚  NARCAN 4mg x 1 |
| ⮚  NTG TABS 0.4mg |
| ⮚  ROCURONIUM 50mg X 2 (60 DAY EXP) |
| ⮚  SUCCS 200mg X 2 (30 DAY EXP) |
| ⮚  VECURONIUM 10mg X 2 |
| ⮚  ZOFRAN 4mg X 2 |

Medical flight company secondary bag

| ⮚  10CC, 3CC, 1CC and 10CC NS FLUSH SYRINGES x 3 EACH  ⮚  NS 250CC x 1, 100CC x 2  ⮚  D5W 250CC x 1 | |
| --- | --- |
| ⮚  AMIODARONE 150MG x 3 | |
| ⮚  CALCIUM GLUCONATE 1G x 1 | |
| ⮚  DILANTIN 250MG x 4 | |
| ⮚  DILTIAZEM 125MG | |
| ⮚  DOBUTAMINE 250MG x 1 | |
| ⮚  DOPAMINE 400MG x 1  ⮚  EPI 1:1000 30 ML x 1 (MULTIDOSE) | |
| ⮚  ESMOLOL PREMIX 2.5G/250CC x 1 | |
| ⮚  K ACETATE x 1, K PHOS x 1 | |
| ⮚  LABETALOL 100MG x 1 | |
| ⮚  LASIX 100MG x 1 | |
| ⮚  LIDOCAINE PREMIX 2G/250CC x 1 | |
| ⮚  MAGNESIUM SULPHATE 5G x 2 | |
| ⮚  NAHCO3 4.2% x 1 | |
| ⮚  NAHCO3 8.4% x 1 | |
| ⮚  NEB MEDS: | |
| o   ALBUTEROL MDI | |
| o   ALBUTEROL MULTI DOSE | |
| o   ATROVENT FISH x 3 | |
| o   DROPPER | |
| o   RACEMIC EPI x 3 | |
| ⮚  NTG GTT 50MG/250CC IN NEOPRENE HOLDER | |
| ⮚  PHENYLEPHRINE 10MG x 5 | |
| ⮚  PITOCIN 10U x 3 | |
| ⮚  PROCAINAMIDE 1G x 2 | |
| ⮚  SOLU-MEDROL 125MG x 1 | |
| ⮚  TERBUTALINE 1MG x 5 | |
| ⮚  TXA 1 G x 2  ⮚  VASOPRESSIN 20U x 5 | |

| Medical flight company Narcotics pack  ⮚  MORPHINE 10MG x 3  ⮚  FENTANYL 100MCG x 3  ⮚  ATIVAN 2MG x 3  ⮚  MIDAZOLAM 5MG x 3  ⮚  KETAMINE 100MG x 2 |
| --- |


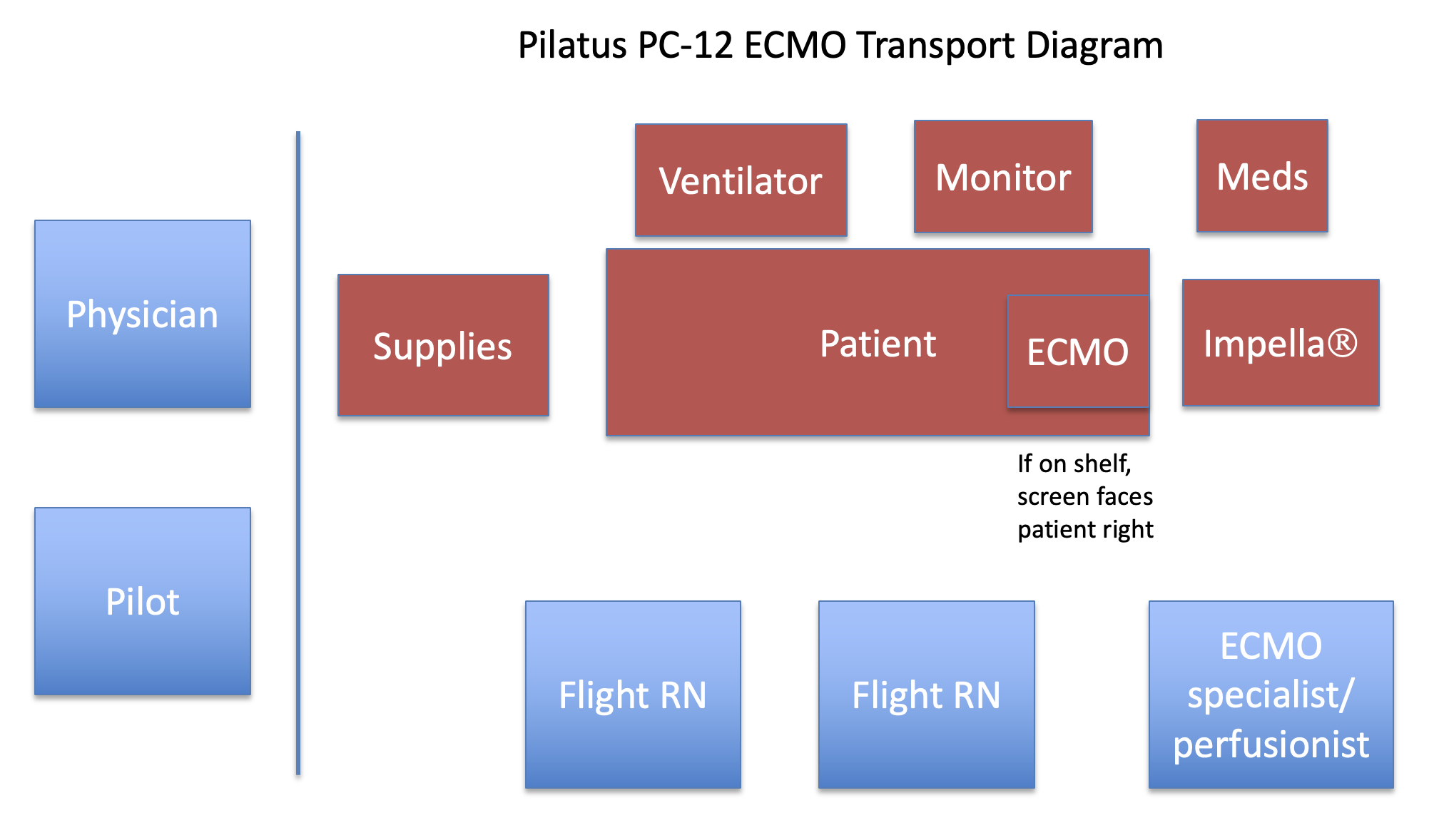

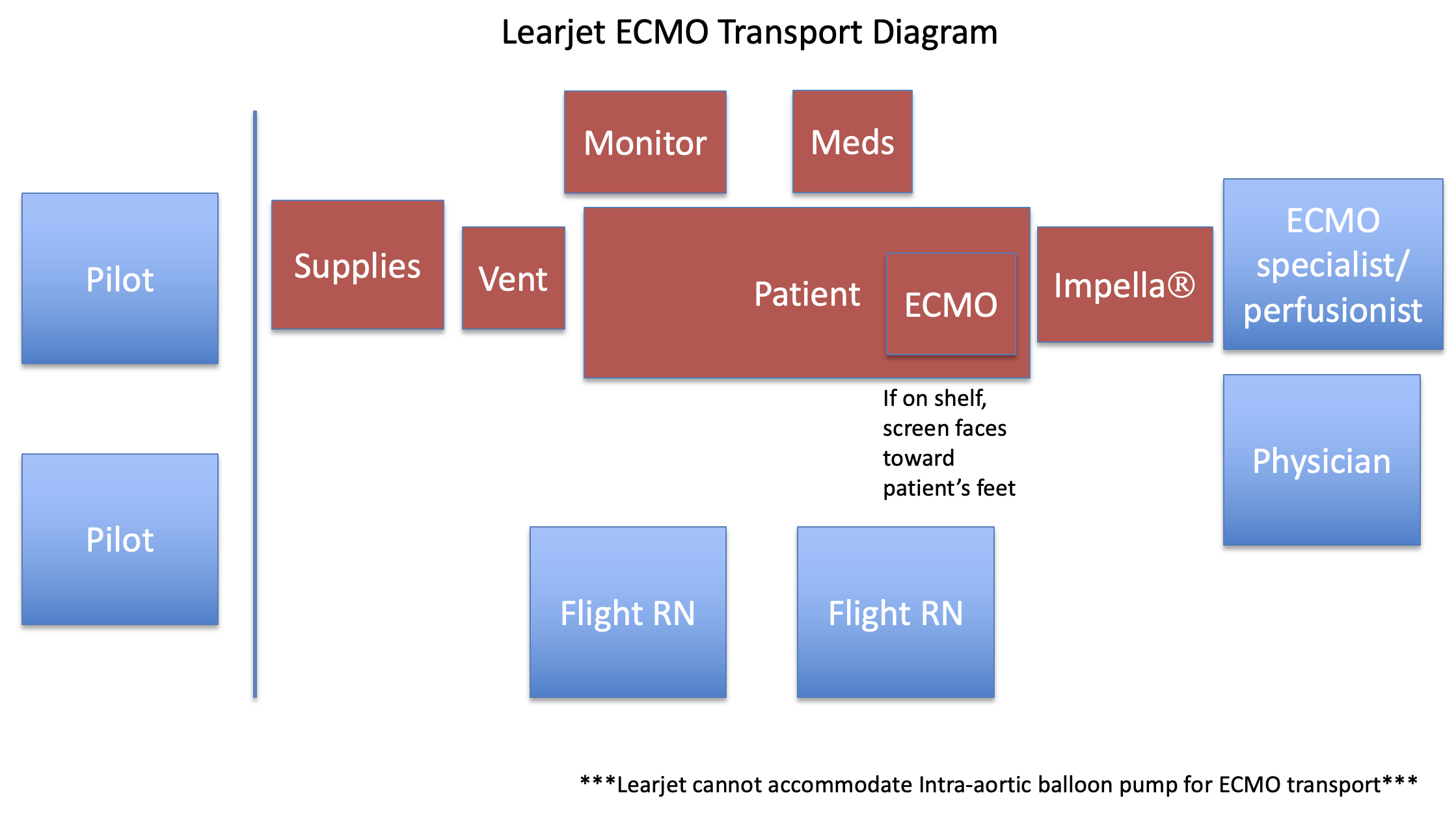


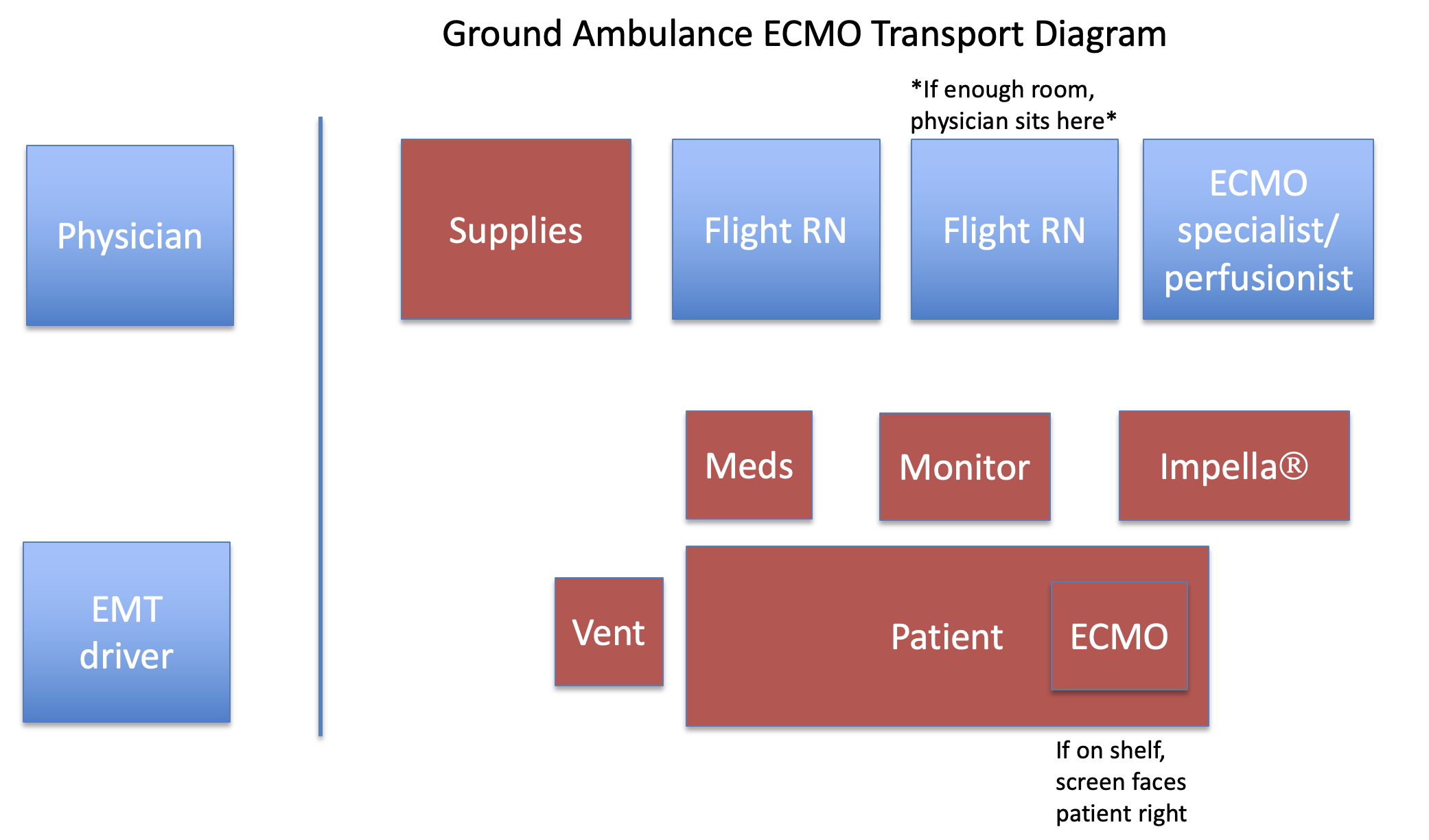


**Referring Hospital Checklist for Secondary ECMO Transport**

**(patient placed on ECMO at Outside Hospital)**

**Heparin Requirement:**

- Heparin 1,000 U/ml 30ml (In syringe)

**IV Drip Requirements:**

- Please prepare an extra bag (or 2 depending on length of transport) of all vasoactive medications and heparin running in drip form
- Please prepare extra bags of narcotic drips (note: we cannot transport using a PCA pump)

**Personnel Requirements:**

- Physicians capable of performing/assisting with circuit change

**Patient Requirements**

- Adequate IV access including secure central IV access
- Well-secured ECMO cannulas

**Referring Hospital Checklist for Primary ECMO Transport**

**(ECMO Hospital places patient on ECMO at Outside Hospital)**

**Heparin Requirement:**

- Heparin 1,000 U/ml 30ml (In syringe)

**IV Drip Requirements:**

- Please have an extra bag (or 2 depending on length of transport) of all vasoactive medications and heparin running in drip form
- Please have extra bags of narcotic drips (note: we cannot transport using a PCA pump)

**Patient Requirements**

- Secure central IV access (not in the R IJ for primary transport for VV ECMO)
- (Primary transport: Patient cannot have IVC filter in place because unable to place drainage cannula in IVC)

**Personnel Requirements:**

- 1 Physician familiar with central line insertion to assist with cannulation
- 1 nurse or scrub tech familiar with sterile technique to assist with hand-off of sterile supplies

**Equipment Requirements:**

- Portable ultrasound with vascular and phased array (cardiac) probe
- Fluoroscopy or TEE (plus physician operator) or portable chest xray: for cannula location verification
- Mayo stand/table

**Blood bank Requirements:**

- 2 units PRBC
- 2 units FFP
- 1 platelet apheresis unit
- Please plan to have blood to bedside upon team arrival. May require more products if bleeding.
- We will bring the cooler, please provide ice

**ECMO Transport Checklists**

ECMO specialist/perfusionist and physician meet at ECMO Hospital to gather supplies

**Pre-Departure at ECMO Hospital**

**Physician**

- Obtain existing patient information from accepting doctor
- Outline plan for patient care with specialist/perfusionist
- Review cannulation/circuit change plan- additional supplies needed?
- Review medication plan- additional medications needed that referring hospital doesn’t have?
- see “ECMO Medication List” for medications already included in medical flight company protocol

**Specialist/Perfusion**

- Gather ECMO transport equipment and recheck checklist in ECMO supplies bag

| **HMC/Montlake Equipment**:   - ECMO supplies bag - Cardiohelp pump, power cord, hand crank - Hemochron®, 10 cuvettes - (Impella® or intra-aortic balloon pump console) - Inhaled epoprostenol? - Any additional meds/drips that referring hospital can’t provide prepared in bags? dexmedetomidine, antibiotics, propofol, nitroprusside, etc |
| --- |
| **Personal Supplies**:   - Cell phone charger, sunglasses, food, personal protective equipment |

ECMO specialist/perfusionist and physician transport from ECMO hospital to aircraft hangar

**Pre-Departure in Hangar**

**Physician**

- Through transfer center, get referring hospital bedside nurse & specialist/perfusionist (+/- physician) report on speaker phone with specialist/perfusion & flight RNs
  - Ensure all relevant information on “ECMO Patient Data Sheet” obtained
  - Review “Referring Hospital Checklist” with refering hospital
- Outline plan for patient care with whole team
- Flight suit

**Specialist/Perfusion**

- Flight suit

**Flight RN**

- Gather medical flight company equipment

| **Medical flight company equipment**   - adult ECMO bag - Transport bags (primary and secondary ICU bag) - (intra-aortic balloon pump/Impella® bag) - Narcotics - i-STAT (handheld aboratory blood analyzer) - Blood cooler (2 packed red blood cells, 2 fresh frozen plasma) - Charting laptop - Additional IV pumps (recommend 6-8 total) - Lifepack 15 (monitor), 2 extra batteries - Ventilator - Vital signs roll/cables/monitoring devices - 2 liters crystalloid - Oxygen tank (total 2 full tanks, split regulator) - Heating blanket (additional for cold weather) - (Intra-aortic balloon pump console) |
| --- |

Team travels to Referring Hospital

**Referring Hospital Arrival**

**Order of operations (see below for team member roles):**

1. Bedside handoff
2. Patient evaluation/exam
3. Cannulation or circuit change
4. (Change over Impella®/intra-aortic balloon pump)
5. Change over medications & ventilator
6. Patient transfer onto flight stretcher
7. Referring hospital departure

**Physician**

- Lead bedside handoff:
  - Time out: Identify patient
  - Referring hospital physician report
  - Referring hospital RN report
  - Referring hospital RT report
  - (Referring hospital perfusion/specialist report)

**Patient evaluation/exam**

**Physician/specialist/perfusion/light RN**

- - Physical exam
  - Ventilator settings, endotracheal tube size/depth
  - Medications/infusions
  - IV access, additional access needed?
  - Other tubes/lines/drains
  - Urine output
  - Review patient’s chart
    - Past medical history (verify with family)
    - Imaging
    - Labs
  - (ECMO settings, cannulas, distal perfusion cannula & limb perfusion)
  - (Impella®/intra-aortic balloon pump settings)

**Pre-Circuit Change (or Cannulation)**

**Physician**

- (Consent obtained for cannulation)
- (Arrange for imaging: fluoroscopy, ultrasound, trans-esophageal echocardiogram, x-ray)
- Outline plan for procedure
  - Circuit change plan

or

- - (Cannulation configuration, verify available equipment)
- Heparin plan?
- (Blood products?)
- (Left ventricular vent plan?)
- (Limb perfusion plan?)
- Lead *Time out* prior to cannulation or circuit change– everyone paying attention, briefly describe plan & assign roles:
- Person and assistants performing circuit change/cannulation
- Referring hospital or ECMO physician managing patient: vasoactive medications (drips, push dose pressors?, calcium?) and rescue ventilator settings
- Referring hospital nurse adjusting medications
- Referring hospital respiratory therapy adjusting ventilator
- Specialist/perfusionist running the pump

**Specialist/perfusion**

- Outline circuit change plan

or

- (New circuit primed, ready to hand-off circuit)

**Impella® or Intra-aortic balloon pump change**

**Specialist/perfusion**

- Intra-aortic balloon pump checklist
- Pressurized crystalloid bag de-aired and full
- Helium tank & batteries full
- Pneumatic leak test completed
- Proper placement verified (chest x-ray)
- No blood in helium driveline
- Impella® checklist
- Verify Tuohy-Borst is tight (compression valve at hub)
- Extra Impella® purge solution, extra purge cassette
- Pressurized 0.9% NS bag connected to red port of Impella® catheter (2.5 and CP only)
- Purge solution connected to yellow port of Impella® catheter (D5 with 50 units heparin per mL)
- Position verified on echocardiogram

**Change over medications & ventilator**

**Flight RN**

- Change over medications
- Change over ventilator
- (change over inhaled epoprostenol)

**Patient Transfer (EVERYTIME onto/off stretcher, into/out ambulance/aircraft)**

**Physician**

- Lead *Time out* – everyone paying attention, briefly describe plan for move – anyone may call STOP
- Move location ready
- Airway (identify watcher, bag valve mask)
- ECMO cannulas (identify watcher)
- Monitor (identify watcher)
- IV access (identified and available)
- Lines, cables, hoses (clear and ready)
- Safety belts (ready for move)
- Power cords easily accessible for early connection
- Oxygen easily accessible for early connection

**Referring Hospital Pre-Departure**

**Physician**

- Lead review of **patient parameters:**
- Hemodynamics, gas exchange, anticoagulation adequate
- Labs checked
- IV access: lines labeled, emergency IV access identified
- Correct medication drips infusing, clamps open
- Blood tubing primed with crystalloid- available for fluid bolus or product
- Bag valve mask available
- Outline hemodynamic goals
- Plan if need to emergently come off ECMO
  - Circuit emergencies plans (air, decannulation, circuit rupture/damage, pump failure)
  - Patient management plan
- Emergency supplies easily accessible (in pocket):
  - Flight RN: code medications, push dose pressors, calcium, etc?
  - Perfusionist/specialist: clamps, 60cc syringe, priming line, crystalloid, etc?
  - Physician: suture, etc?
- (Inhaled epoprostenol running and appropriately connected)
- Lead review of **ECMO parameters:**
- Cannula secure
- Cannula position verified on echo/chest x-ray
- Limb perfusion adequate
- Left ventricular vent adequate, Impella®/intra-aortic balloon pump settings adequate
- Alarm parameters set and alarms on
- Blood flow appropriate
- Gas flow connected properly, normal tubing color change
- Heater on and temperature set, keep plugged in until immediately before departure, assess need for heating blanket (wrap patient & tubing for cold weather)
- Ensure deairing cap **ON** for all transport and bed transfers (risk of de-priming membrane lung with elevation relative to patient)

| ECMO Hospital equipment   - ECMO supplies bag - Cardiohelp hand crank, power cord - (Impella® console) |
| --- |
| Medical flight company equipment   - Medical flight company transport bags (primary and secondary ICU bag) - ECMO bag - (Intra-aortic balloon pump/Impella® bag) - iSTAT hand held laboratory blood analyzer - Lifepack 15 monitor with cables attached to patient - Charting laptop - Narcotics - Extra medication pumps - Oxygen tanks - Additional IV pumps - Oxygen tank (total 2 full tanks, split regulator) - (Intra-aortic balloon pump console) |
| New supplies   - Additional blood from referring hospital in medical flight company cooler - Additional medications & fluids from referring hospital - Patient records/chart, imaging |

Team transports patient to ambulance/aircraft

**Patient Transfer (EVERYTIME onto/off stretcher, into/out ambulance/aircraft)**

**Physician**

- Lead *Time out* – everyone paying attention, briefly describe plan for move – anyone may call STOP
- Move location ready
- Airway (identify watcher, bag valve mask)
- ECMO cannulas (identify watcher)
- Monitor (identify watcher)
- IV access (identified and available)
- Lines, cables, hoses (clear and ready)
- Safety belts (ready for move)
- Power cords easily accessible for early connection
- Oxygen easily accessible for early connection

**Once inside ambulance/aircraft**

**Physician**

- Lead review:
- Oxygen flow on & tank open
- Ventilator (connected to wall oxygen)
- ECMO pump (gas connected, plugged in)
- (Impella®/intra-aortic balloon pump plugged in)
- Heating blanket in place
- Vital signs (monitor visible)
- IV infusions (running at correct rate)
- Recheck all straps and belts – equipment is secured

**Flight RN**

- Emergency equipment accessible
- Patient data charting
- Call ahead to ECMO hospital for nursing report (to have infusing medications ready)

**Specialist**

- Emergency equipment accessible
- Hand crank in place
- ECMO pump charting
- (Impella®/intra-aortic balloon pump charting)

Team arrives at ECMO Hospital with patient

**Patient Transfer (EVERYTIME onto/off stretcher, into/out ambulance/aircraft)**

**Physician**

- Lead *Time out* – everyone paying attention, briefly describe plan for move – anyone may call STOP
- Move location ready
- Airway (identify watcher, bag valve mask)
- ECMO cannulas (identify watcher)
- Monitor (identify watcher)
- IV access (identified and available)
- Lines, cables, hoses (clear and ready)
- Safety belts (ready for move)
- Power cords easily accessible for early connection
- Oxygen easily accessible for early connection

**Bedside Handoff at ECMO Hospital**

**Physician**

- Lead bedside handoff:
  - - Time out: Identify patient
    - Physician report: brief overview of patient and transport course
    - Flight RN report: review of tubes/lines/drains, medication administered, iSTAT labs obtained, ventilator settings
    - Specialist/perfusion report: ECMO settings, cannula size and depth

**Post Transport**

**Physician**

- Transport note in Epic (include referring doctor’s name): brief description of transport events
- Cannulation note in Epic
- Debrief form completed and turned in to ECMO coordinator

**Specialist/perfusion**

- ECMO (and Impella®/intra-aortic balloon pump) charting to be given to ECMO hospital bedside specialist and make copy to include in medical transport company report
- Surgical instrument tray to sterile processing
- ECMO hospital transport bag restocked
- Hemacron data downloaded and returned

**Flight RN**

- Report scanned into electronic medical record

Team transported back to aircraft hangar in ambulance

**ECMO Transport Debrief: To be led by ECMO physician with all team members present**

Transport Date: ________________________________

Patient Name: ________________________________

Referring Hospital: ________________________________

ECMO Hospital: ________________________________

| Delays |  |
| --- | --- |
| Deficiencies/ missing supplies |  |
| Transport logistical problems |  |
| Equipment problems |  |
| Adverse events |  |

**ECMO physician: ______________________________**

**ECMO physician: ______________________________**

**Specialist/perfusionist: ______________________________**

**ALNW RN: ______________________________**

**ALNW RN: ______________________________**

Return to ECMO coordinator upon completion
