# Supplementary material for: A Simulation Curriculum for Ground and Air ECMO Transport
Source: MedEdPORTAL. 2025 Mar 18;21:11508. doi: 10.15766/mep_2374-8265.11508 (PMC11913753; doi:10.15766/mep_2374-8265.11508)
Supplement: Supplementary file 1 — ECMO Transport Protocol.docxECMO Transport Logistics and Emergency Simulations.docxECMO Transport Needs Assessment.docxECMO Simulation Images.docx [file mep_2374-8265.11508-s001.zip › C. ECMO Transport Needs Assessment.docx]

Appendix C: ECMO Transport Curriculum Needs Assessment

Description: This document can be used by programs to perform a needs assessment of their current program and personnel.

**ECMO Transport Curriculum Needs Assessment**

1. What is your current position?
   1. Physician
   2. Perfusionist
   3. ECMO RN specialist
   4. Airlift NW Nurse
2. Approximately how many ECMO transports have you done in your career?
   1. 0-5
   2. 6-10
   3. 11-15
   4. 16-20
   5. 21+
3. How strongly do you agree with the following statement: "I currently feel confident in my ability to transport an ECMO patient?”
   1. Likert Scale (Strongly disagree – Disagree – Neutral – Agree – Strongly Agree)
4. How strongly do you agree with the following statements regarding logistics of air and ground ECMO patient transport: “I feel prepared within my role on the ECMO team regarding...”? (Likert Scale used for all responses)
   1. Equipment availability and accessibility during transport
   2. Medication availability and accessibility
   3. Physical loading and unloading of patients from the ambulance and aircraft
   4. Implementation and usage of ECMO transport protocols and checklists
   5. Incorporating principles of flight physiology into the care and management of a patient receiving ECMO
   6. Pre-flight takeoff procedures and in-flight communication standards
   7. Emergency procedures in-flight (I.e. aircraft shut-down, location/use of fire extinguishers, emergency egress, Emergency Locator Transmitter (ELT) activation, use of radios, orientation to survival kit
5. How strongly do you agree with the following statements: “During ECMO transport, I feel prepared for my role in the management of...” (Likert Scale used for all responses)
   1. Air entrainment
   2. Pump failure/hand cranking
   3. Circuit disruption (i.e., broken pigtail) and need for component replacement
   4. Circuit change
   5. Cardiac arrest
   6. Accident decannulation
   7. Hypotension
   8. Hypoxemia
   9. Bleeding
6. Which of the following topics do you feel are the most important to cover during the ECMO transport simulation? (Select top three choices)
   1. Air entrainment
   2. Pump failure/hand cranking
   3. Circuit disruption (i.e., broken pigtail) and need for component replacement
   4. Circuit change
   5. Cardiac arrest
   6. Accident decannulation
   7. Hypotension
   8. Hypoxemia
   9. Bleeding
7. In your opinion, how often should you either participate in an ECMO transport simulation OR transport an ECMO patient to ensure maintenance of skills?
   1. 0-3 months
   2. 3-6 months
   3. 7-12 months
   4. 12-24 months
8. Have you participated in UW/ALNW Transport Team simulation exercise yet?
   1. Yes
   2. No
9. If you have participated in the ECMO transport simulation, what (if anything) would you change about it to better target your learning needs? What parts were the most helpful and which parts could be cut out?
   1. (Short answer/open ended responses allowed here, but no response required)
10. What other general education would you like to augment your skills as a member of the ECMO Transport Team? (Able to choose more than one, but no response required)
    1. Didactics on ECMO management/physiology
    2. Didactics on ECMO emergencies/troubleshooting
    3. Water drills and physical manipulation of the circuit for air entrainment, hand cranking, etc.
    4. Other (short answer/open ended responses allowed here)

**ECMO Transport Curriculum Post Assessment**

1. How strongly do you agree with the following statement: "I currently feel confident in my ability to transport an ECMO patient."
   1. Likert Scale (Strongly disagree – Disagree – Neutral – Agree – Strongly Agree)
2. How strongly do you agree with the following statement: "After the simulation, I feel that I understand my role on the ECMO transport team"
   1. Likert Scale (Strongly disagree – Disagree – Neutral – Agree – Strongly Agree)
3. How strongly do you agree with the following statements regarding logistics of air and ground ECMO patient transport: "I feel prepared within my role on the ECMO team regarding..." (Likert Scale used for all responses)
   1. Equipment availability and accessibility during transport
   2. Medication availability and accessibility
   3. Physical loading and unloading of patients from the ambulance and aircraft
   4. Implementation and usage of ECMO transport protocols and checklists
   5. Incorporating principles of flight physiology into the care and management of a patient receiving ECMO
   6. Pre-flight takeoff procedures and in-flight communication standards
   7. Emergency procedures in-flight (i.e. aircraft shut-down, location/use of fire extinguishers, emergency egress, Emergency Locator Transmitter (ELT) activation, use of radios, orientation to survival kit)
4. How strongly do you agree with the following statements "During ECMO transport, I feel prepared for my role in the management of..." (Likert Scale used for all responses)
   1. Air Entrainment
   2. Pump Failure/Hand Cranking
   3. Circuit disruption (i.e., broken pigtail) and need for component replacement
   4. Circuit change
   5. Cardiac arrest
   6. Accident decannulation
   7. Hypotension
   8. Hypoxemia
   9. Bleeding
5. What (if anything) would you change about the ECMO Transport simulation to better target your learning needs? What parts were the most helpful and which parts could be cut out? (Short answer/open ended responses allowed here)
