# Supplementary material for: A Simulation Curriculum for Ground and Air ECMO Transport
Source: MedEdPORTAL. 2025 Mar 18;21:11508. doi: 10.15766/mep_2374-8265.11508 (PMC11913753; doi:10.15766/mep_2374-8265.11508)

Appendix D: ECMO Retrieval Team Simulation Images

Description: This document is a composition of several images to help visualize the components of our simulation which include the hospital room, ground and air transports spaces, and the high fidelity ECMO emergency scenarios to practice.

Location #1: Simulated “Referring Hospital ICU” & “Receiving Hospital ICU”


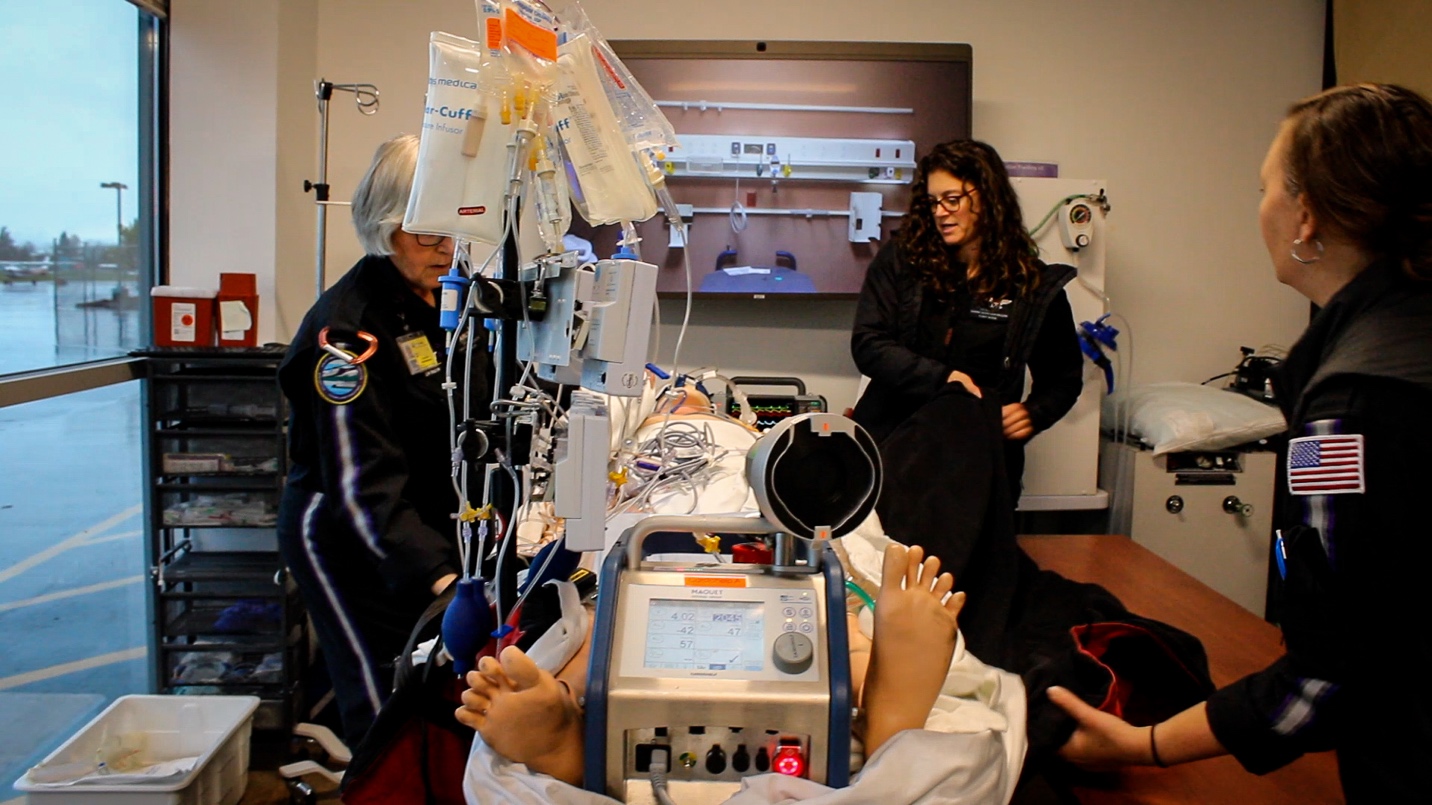


Location #2: Ambulance


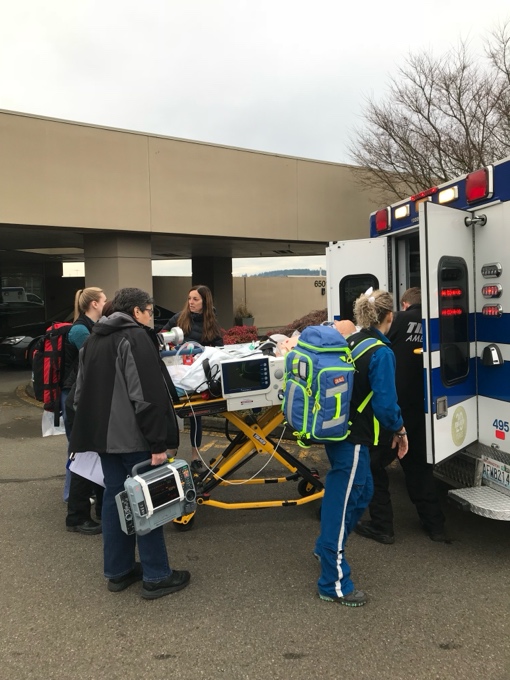

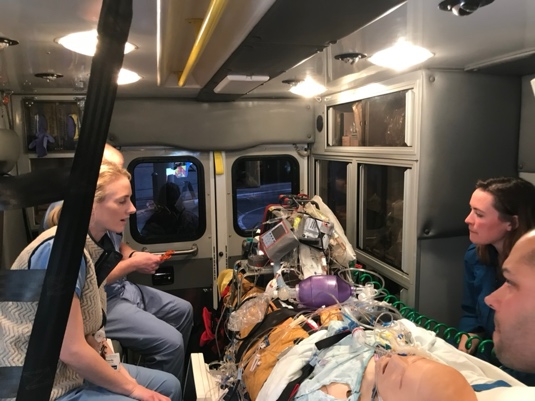


Location #3: Aircraft


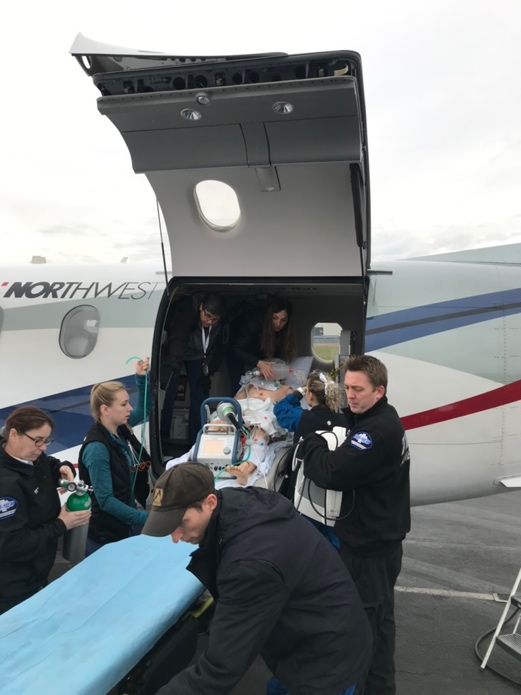

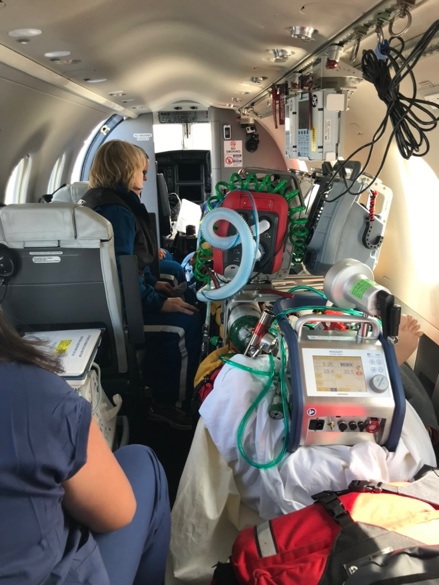


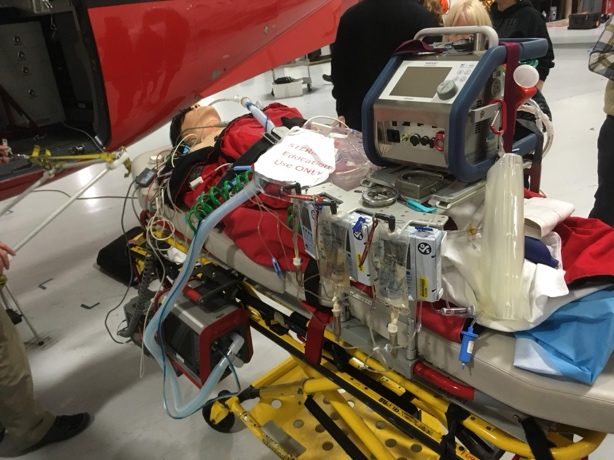


Scenario A: Pump failure


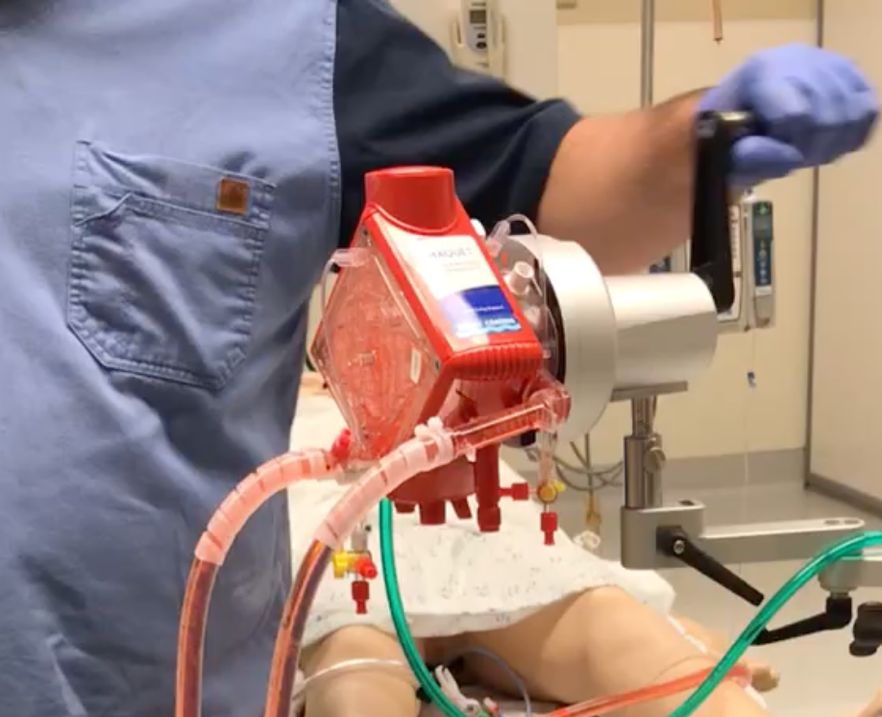


Scenario B: Air entrainment


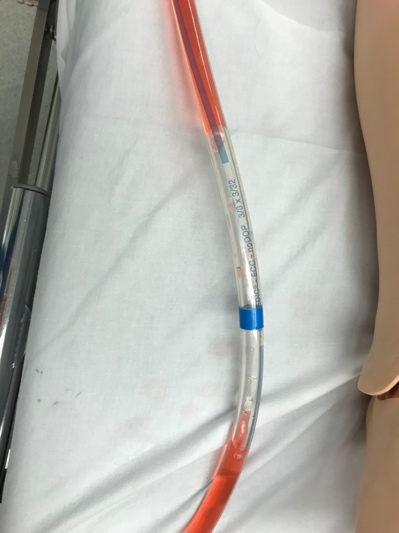

Supplement: Supplementary file 1 — ECMO Transport Protocol.docxECMO Transport Logistics and Emergency Simulations.docxECMO Transport Needs Assessment.docxECMO Simulation Images.docx [file mep_2374-8265.11508-s001.zip › D. ECMO Simulation Images.docx]
