# Supplementary material for: A Simulation Curriculum for Ground and Air ECMO Transport
Source: MedEdPORTAL. 2025 Mar 18;21:11508. doi: 10.15766/mep_2374-8265.11508 (PMC11913753; doi:10.15766/mep_2374-8265.11508)
Supplement: Supplementary file 1 — ECMO Transport Protocol.docxECMO Transport Logistics and Emergency Simulations.docxECMO Transport Needs Assessment.docxECMO Simulation Images.docx [file mep_2374-8265.11508-s001.zip › B. ECMO Transport Logistics and Emergency Simulations.docx]

| **Appendix B: Simulation Case**  **SIMULATION CASE TITLE:** ECMO Transport Logistics and Emergency Simulations  **AUTHORS:** Shouldice, Felix, Danielson, Plourde, Johnson, Latimer, Utarnachitt, Badulak | |
| --- | --- |
| **PATIENT NAME:** Jane Smith  **PATIENT AGE:** 40 F  **CHIEF COMPLAINT:** Cardiogenic Shock on VA ECMO | |
|  | |
| **Brief narrative description of case**  *Include the presenting patient chief complaint and overall learner goals for this case* | A patient is in critical cardiogenic shock at a referring hospital, was stabilized with VA ECMO cannulation and the ECMO transport team is called for transfer. The team will demonstrate appropriate use of protocols and checklists throughout the scenario as well as clinical skills for emergencies encountered.  The physician and perfusionist/specialist get patient report through the transfer center and gather supplies at the receiving hospital then meet up with the flight nurses in the aircraft hangar to gather additional supplies and review transport plan.  The team arrives in the referring hospital ICU and receives patient hand-off, performs circuit change and departs referring hospital.  The team loads patient into the ambulance where they encounter clinical emergencies. First pump failure will occur; the team must demonstrate separation from ECMO, use of hand-crank and re-initiation of blood flow. Second, air entrainment will occur and the team must demonstrate separation from ECMO, de-airing of circuit, supporting patient off ECMO and re-initiation of blood flow.  Subsequently the team will unload the patient from the ambulance, load and unload into an aircraft and end in the receiving hospital’s ICU. A patient handoff will occur and the simulation will end. |
| **Primary Learning Objectives**  *What should the learners gain in terms of knowledge and skill from this case? Use action verbs and utilize Bloom’s Taxonomy as a conceptual guide* | By the end of this activity, learners will be able to:   1. Demonstrate appropriate use of the ECMO transport protocol and checklists. 2. Exhibit safe ambulance and aircraft loading and unloading practices. 3. Recognize and demonstrate the appropriate corrective steps for pump failure during ECMO transport. 4. Recognize and demonstrate the appropriate corrective steps for air entrainment during ECMO transport. |
| **Critical Actions**  *List which steps the participants should take to successfully manage the simulated patient. These should be listed as concrete actions that are distinct from the overall learning objectives of the case.* | Protocol/Checklist   1. Complete steps within protocol for each phase of transport:    1. Receiving hospital    2. Hangar    3. Referring hospital handoff    4. Aircraft/ambulance    5. Receiving hospital hand off 2. Before any patient transfer a timeout and role assignment is utilized   Pump Failure Simulation – Simulation A   1. Communicate need to support patient off ECMO to the team 2. Separate the patient from the ECMO circuit (using the correct order of clamping; arterial then venous) 3. Transfer the pump head to hand crank 4. Correctly re-initiate ECMO (spin the hand crank, correct order of clamp removal- venous then arterial, increase RPM’s to previous speed)   Air Entrainment Simulation – Simulation B   1. Identify air within the ECMO circuit 2. Vocalize need to determine source of air 3. Separate the patient from the ECMO circuit if airlock or arterial air (using the correct order of clamping; arterial then venous, decrease speed to zero) 4. Communicate need to support patient off ECMO to the team 5. De-air the ECMO circuit 6. Correctly re-initiate ECMO (increase RPM’s, correct order of clamp removal- venous then arterial, arterial bubble alarm armed, increase RPM’s to target flow rate) |
| **Learner Preparation**  *What information should the learners be given prior to initiation of the case?* | The simulation participants will be given the following documents prior to the simulation:   - ECMO transport protocol - ECMO transport equipment checklist |

| Initial Presentation | | | |
| --- | --- | --- | --- |
| **Initial vital signs** | T: 37.0 HR: 115 BP: 75/65 O2: 100% Vent: RR: 10 PEEP: 5 FiO2: 40% TV: 8cc/kg | | |
| **Initial ECMO settings** | RPM: 3200, Flow 4 L/min, Pven -80 mm Hg, Part 250 mm Hg, delta P 25 mm Hg  Sweep: 2 L/min, FdO_2_ 100% | | |
| **Overall Appearance**  *What do learners see when they first enter the room?* | There are multiple settings for this simulation in order to accurately portray the various stages of ECMO transport.  Location #1 – Aircraft Hangar  Location #2 – ICU room – Receiving/Referring Hospital  Location #3 – Ambulance  Location #4 – Aircraft  The first two locations (Aircraft Hangar and ICU room) are to simulate using the protocols, checklists, and ensuring that the team has the appropriate equipment that may be necessary.  When they first encounter the patient for the first time, they will walk into a simulated ICU room. There will be a patient who is intubated and on ECMO. Next to the bedside are the ventilator, ECMO pump, arterial line, pulmonary artery catheter, Impella console, and IV poles. | | |
| **Actors and roles in the room at case start**  *Who is present at the beginning and what is their role? Who may play them?* | Actors throughout case:   - Facilitator(s) will simulate:   - Outside hospital MD: gives report on patient presenting complaint, hospital course, ventilator settings, overall status   - Outside hospital bedside nurse: gives report on current vitals, access, equipment, medications   - Outside hospital perfusionist/ECMO specialist: gives report on ECMO circuit and current ECMO settings   Initial team roles at case start:   - Physician (x1) role:   - Patient management decisions   - Leads checklists and time-outs - ECMO specialist/perfusionist (x1) role:   - Responsible for ECMO equipment, bags, circuit   - Manages ECMO circuit, Impella - Airlift critical care nurse (x2) role:   - Responsible for Airlift equipment, ventilator, monitor, IV pumps, iSTAT, blood   - Manage medications, infusions, blood product administration   - Manage ventilator   - Lead patient movement logistics (load/unload/transfer) | | |
| **HPI**  *Please specify what info here and below must be asked vs what is volunteered by patient or other participants* | The transport team is activated to retrieve a patient emergently cannulated this morning for VA ECMO for critical cardiogenic shock from acutely decompensated heart failure due to chronic biventricular non-ischemic cardiomyopathy.  She has required vasopressor support and subsequently had an Impella placed for left ventricular venting. The referring hospital is now requesting transfer to your hospital for bridge to heart transplant. | | |
| **Past Medical/Surgical History** | **Medications** | **Allergies** | **Family History** |
| Non-ischemic cardiomyopathy | ASA  Atorvastatin  Lisinopril  Furosemide  Spironolactone  Carvedilol | NKDA | No alcohol or smoking history |
| **Physical Examination** | | | |
| **General** | Sedated and intubated, height: 5’6”, weight 60 kg, BSA 1.7 kg/m^2^ | | |
| **HEENT** | Endotracheal tube in oropharynx | | |
| **Neck** | Trachea midline | | |
| **Lungs** | Bilateral lungs sounds clear | | |
| **Cardiovascular** | Distant heart sounds, regular, arterial line in place R radial artery | | |
| **Abdomen** | Soft, non-distended | | |
| **Neurological** | Sedated, moves extremities to command | | |
| **Skin** | No rashes, dry | | |
| **GU** | Normal female GU, foley with straw colored urine | | |
| **Psychiatric** | Sedated, unable to assess | | |
| **Additional Data** | ECMO Patient Data Sheet   1. Patient name, age: Jane Brown, 40F 2. Height, weight & BSA: 5’6”, 60 kg, BSA 1.7 kg/m^2^ 3. Referring hospital: American General 4. Name and phone number of referring physician: Dr. Jones 5. General diagnosis and past medical history: chronic biventricular non-ischemic cardiomyopathy 6. Indication for ECMO: cannulated this morning for VA ECMO for critical cardiogenic shock from acutely decompensated heart failure 7. Last ABG: pH_7.35__ PaCO_2_:_39_ PaO_2_:_250__ HCO_3_:_22__ SaO_2_%: _100__ 8. Last BMP: Na 135, K 4.0, Cl 101, HCO3 21, BUN 54, Cr 2.2, Glu 137 9. Last CBC: WBC 18, Hb 9, Hct 28, Plt 190 10. Last LFTs: Tbili 2.0, Dbili 1.0, AST 427, ALT 329, Alp 109 11. Last coags (PTT, INR, ACT): PTT 90, INR 1.6, ACT 180 12. Last vital signs:     1. Heart Rate: 115     2. Blood Pressure: 75/65     3. SpO_2_: 100%     4. Pulmonary artery catheter: RA 5, PA 35/25, CI 1.3, SvO2 79% 13. Mode of oxygen delivery (nasal canal, intubated, etc): intubated 14. Current ventilator settings:     1. Rate: 10     2. Tidal volume or inspiratory pressure: 8 cc/kg IBW     3. FiO_2_%: 40%     4. PEEP: 5 cm H_2_O 15. Current vasoactive / inotropic medications: dobutamine 5 mcg/kg/min, norepinephrine 0.15 mcg/kg/min 16. Current sedation: propofol 40 mcg/kg/min, fentanyl 50 mcg/kg/min 17. Glasgow Coma Scale, recent neuro exam: moves all extremities to command 18. Average hourly urine output, dialysis?: UOP 20cc/hr, no dialysis 19. Current IV access (central and peripheral): right PICC, R IJ PA catheter 20. IVC filter?: No 21. Results of echocardiogram (R or L ventricular dysfunction? Valvulopathy?): Biventricular severe dysfunction and dilation, aortic valve opens with each beat, no aortic insufficiency 22. Impella or IABP? Settings?: Impella CP at P4 with 2 L/min flow 23. If on ECMO:     1. Run start time/date: 0800 today     2. Blood flow & RPM: 4 L/min, 3200 RPM     3. Sweep gas flow rate: 2 L/min, FdO_2_ 100%     4. Circuit pressures: Pven -80 mm Hg, Part 250 mm Hg, delta P 25 mm Hg     5. Cannulation Configuration (VA, VV, VVA, size of cannulas, distal perfusion?): VA, 25 Fr venous drainage in R femoral vein, 18 Fr arterial return in L femoral artery, distal perfusion cannula in R superficial femoral artery     6. LV vent?: Impella     7. Anticoagulation: heparin 19 U/kg/hr | | |

| Instructor Notes - Changes and CASE Branch Points  *This section should be a list with detailed description of each step than may happen during the case. If medications are given, what is the response? Do changes occur at certain time points? Should the nurse or other participant prompt the learners at given points? Should new actors or participants enter, and when? Are there specific things the patient will say or do at given times? There are a few examples given, but it is expected that most cases will have many more changes and potential branch points.* | | |
| --- | --- | --- |
| **Intervention / Time point** | **Change in Case** | **Additional Information** |
| ECMO specialist/perfusionist and physician meet in “Aircraft Hangar” This is location #1.  *15min for this section* | | |
|  |  | *Facilitator introduces simulation: “The goals of this simulation are to demonstrate use of the protocol and checklists, safely transfer the patient, and navigate ECMO emergencies during transport. We will debrief the entire simulation at the end upon return of the patient to the receiving hospital.” “This simulation will involve 4 physical locations and 2 emergency scenarios.”* |
| Pre-Departure at Receiving Hospital  *Physician and perfusionist/specialist meet in aircraft hangar to gather supplies and obtain patient information* | *No direct patient interaction* | *Transfer center (facilitator) provides patient information on “ECMO Patient Data Sheet”*  *-HPI, exam, labs, etc.* |
| ***Critical Action****: Team follows protocol and equipment checklist* |  | *This will focus on obtaining information and acquiring necessary ECMO equipment for the inter-hospital setting.*  *Facilitator will prompt use of protocols/checklists if not expressly stated*  *-ECMO Transport Protocol*  *-ECMO Equipment Checklist*  *-ECMO Patient Data Sheet*  *-Referring Hospital Checklist* |
| ECMO specialist/perfusionist and physician transport to “Aircraft Hangar”. This is location #1.  *15min for this section* | | |
| Pre-Departure in Hangar  *Physician, perfusionist/specialist meet up with flight RNs to gather supplies and review plan of care* | *No direct patient interaction* |  |
| ***Critical Action****: Team uses protocol and equipment checklist* |  | *This will focus on obtaining and acquiring the necessary EMS equipment for the inter-hospital setting.*  *Again, facilitator will prompt the team to follow protocols/checklists if not expressly stated*  *-EMS equipment and medication checklists* |
| *Review plan of care with the entire team* |  | *If plan is not reviewed, prompt the team to do so* |
| Team travels to “Referring Hospital ICU”. This is location #2.  *15min for this section* | | |
| Referring Hospital patient pick-up  *Team arrives at the referring hospital ICU room.* | *First patient interaction*  *Vital signs: T 37.0, BP 75/65, HR 115*  *Ventilator settings: RR: 10 PEEP: 5*  *FiO2: 40% TV: 8cc/kg* | *Order of operations ideal flow:*   1. *Bedside Handoff* 2. *Patient evaluation* 3. *Circuit change* 4. *Impella change* 5. *Medication change* 6. *Ventilator change* 7. *Transfer patient to stretcher* |
| *Transport team gets bedside handoff, patient exam, circuit change, Impella change, medication and ventilator change* | *10 seconds after circuit change started:*  *65/55, HR 125 (if no pre-emptive escalation of hemodynamic support)*  *85/75, HR 115 (if vasopressors/ impella escalated and/or calcium given)* | *Facilitator says: “the patient is getting hypotensive, is there anything you’d like to do?”* |
| ***Critical Action****: Team uses protocol and equipment checklist* |  |  |
| Patient Transfer (EVERYTIME onto/off stretcher, into/out ambulance/aircraft) |  |  |
| *Critical Action: Team uses a time-out* |  | *Facilitator will prompt use of the Patient Transfer checklist if needed*  *Facilitator will also prompt “would you like to assign a team member to watch monitors/cannulas/airway?”* |
| OSH Pre-Departure  *Transport team reviews patient status and equipment prior to leaving referring hospital* |  |  |
| *Critical Action: Team uses protocol and equipment checklist* |  |  |
| Team transports patient from “Referring Hospital ICU” to the ambulance. The ambulance is location #3. Scenarios A and B will occur in the ambulance.  *45min for this section* | | |
| Inside ambulance | Vital signs throughout the simulation | *Note: the use of the Patient Transfer checklist will be emphasized every time the patient is physically transferred* |
| *Scenario A*  *2 minutes after ambulance has begun moving announce pump failure has occurred/turn off the pump* | Time 0 min: BP 75/65, HR 115 | *Facilitator can say “it’s pretty bumpy in here, do you want the ambulance to pull over or keep driving?”* |
| *Critical Action: Perfusionist/specialist communicates the need to support the patient off ECMO*  ***Critical Action****: Separate the patient from the ECMO circuit*  ***Critical Action:*** *Transfer the pump head to the hand crank* | Time 1 min: 55/45, HR 130, SpO_2_ 100%  Time 2 min: 50/40, HR 140  *65/55, HR 120 (if vasopressors/Impella escalated)* | *Change the vital signs, but no prompting required at this time*  *Facilitator: “this patient is hypotensive- is there anything you’d like to do?”*  *-prompting increased vasopressor and Impella support* |
| *Optional: Rescue ventilator settings request* | *If team placed patient on “ultraprotective” ventilator settings: SpO2 70% at 2min*  *SpO2 100% (if increased to normal tidal volume/inspiratory pressure)* | *Facilitator may prompt “the patient is getting hypoxemic, is there anything you’d like to do?”* |
| *Optional: CPR request* | *Time 4 min: 40/30, HR 150* | *This will occur at 4 minutes into the case if not transferred to hand crank, regardless of giving increased vasopressors*  *Facilitator may prompt “do you want to start CPR?”* |
| ***Critical Action:*** *Correctly re-initiate ECMO* | *If done correctly:*  *Patient vitals: 75/65, HR 115 (starting vital signs)* | *This must be done in the correct order*   1. *Spin hand crank* 2. *Clamp removal (venous then arterial)* 3. *Increase RPM’s to previous speed*   *When done correctly, Scenario A will end* |
| *Allow for 1-2 minutes in between Simulation A and Simulation B* |  | *Facilitator will inform team: “Scenario A has ended please remove pump head from hand crank and re-seat in pump”. Team may quickly debrief among themselves; they will not be prompted to do so.* |
|  |  |  |
| ***Scenario*** ***B***  *Approximately 1 minute after the ECMO pump has been re-seated and supplies have been replaced by the team the second scenario will begin* |  | *The ambulance will begin driving again* |
| *To start the scenario, the facilitator will inject a large amount of air into the ECMO circuit* | *Vital Signs throughout the case* |  |
| ***Critical Action:*** *Identify that there is air within the ECMO circuit* | *Time 0 min: 75/65, HR 115* | *Facilitator can say “it’s pretty bumpy in here, do you want the ambulance to pull over or keep driving?”* |
| ***Critical Action****: Voice need to Identify source of the air* | *Vital signs remain the same* | *This is an important step which must always be done. If not done, more air is injected into the circuit upon re-initiation of ECMO. Facilitator prompts “***looks like there is more air getting into the circuit, do we know where it is coming from?”**  *Once the question is asked, the facilitator will respond with “a dialysis cap was previously left open; it has now been closed” and no further air entrainment will occur* |
| ***Critical Action****: Separate the patient from the ECMO circuit if airlock or arterial air*  ***Critical Action****: Communicate the need to support the patient off ECMO to the team* | Time 1 min: 55/45, HR 130, SpO2 100%  Time 2 min: 50/40, HR 140  *65/55, HR 120 (if vasopressors escalated)* | *These steps need to be done together if the air entrainment is big enough requiring separation from ECMO*  *This will need to be done with the correct order of clamping (arterial then venous), decreasing RPMs to zero*  *When being supported off of ECMO the team may again ask for escalating vasopressors for support* |
| ***Critical Action****: De-air the ECMO circuit* |  | *This will need to be done to effectively resolve airlock and remove any arterial air* |
| *Optional:* | *Time 4 min: 40/30, HR 150, SpO2* | *This will occur at 4 minutes into the case if the patient is not yet back on ECMO, regardless of giving increased vasopressors*  *Facilitator may prompt “do you want to start CPR?”* |
| ***Critical Action:*** *Re-initiate ECMO* | *Vitals: 75/65, HR 115* | *The correct order of re-initiation of ECMO after the air is removed:*   1. *Increase RPM’s* 2. *Release clamps* 3. *Re-arm arterial bubble alarm* 4. *(Be ready to re-clamp if bubbles visualized)* 5. *Continue increasing RPM’s to target flow rate* |
| *Allow for 1-2 minutes after Scenario B before resuming patient transport* |  | *Facilitator will inform team: “Simulation B has ended.” Team may quickly debrief among themselves; they will not be prompted to do so.* |
| **Team transports the patient from the ambulance into and out of the aircraft. This is location #4.**  *15min for this section* | | |
| Patient Transfer (EVERYTIME onto/off stretcher, into/out ambulance/aircraft) |  |  |
| ***Critical Action****: Team uses a time-out* |  | *Facilitator will prompt use of the Patient Transfer checklist if needed*  *Facilitator will also prompt “would you like to assign a team member to watch monitors/cannulas/airway?”* |
|  |  |  |
| Team transports the patient from the aircraft back to the “Receiving Hospital ICU”. This is from location #4 to location #2.  *15min for this section* | | |
| Patient Transfer (EVERYTIME onto/off stretcher, into/out ambulance/aircraft) |  |  |
| ***Critical Action****: Team uses a time-out* |  | *Facilitator will prompt use of the Patient Transfer checklist if needed*  *Facilitator will also prompt “would you like to assign a team member to watch monitors/cannulas/airway?”* |
| Debrief: Debrief simulation including use of protocols, patient transfers, pump failure and air entrainment emergencies | | |

Additional information

- 1. Ideal Scenario Flow
     1. *The overall goal of the scenario is to help implement and teach an ECMO patient transport curriculum using the aid of standardized checklists and protocols as well as prepare healthcare providers for some of the possible ECMO emergencies and how to respond to them appropriately while in the inter-hospital setting.*
     2. *Ideally the team utilizes the provided protocols and checklists without prompting throughout the initial stages of gathering the materials at the hospital and at the airlift hanger and familiarizes themselves with the location of all these things within their gear in case it is needed. Once the team arrives at the outside hospital ICU room and meets the patient, they will need to disconnect the patient from the hospital’s equipment, connect the patient to their equipment, change the circuit, and then transfer her to their stretcher and then to the ambulance. The circuit change must be done expeditiously or the patient will become hypotensive. All of the steps have been listed for them in the protocols that they will have been given, but given the complex and tenuous nature of patients on ECMO, they will be prompted to continually use the checklists in order to not overlook steps in transport.*
     3. *Once the patient has been successfully transferred into the ambulance, 2 different scenarios challenge the team to navigate emergencies during transport. The first will involve pump failure. The team will need to recognize this immediately, clamp the circuit (taking the patient off ECMO support) and then transfer the pump itself to the hand-crank, spin the crank and unclamp in correct order to manually re-initiate ECMO support. This should be done expeditiously, as the patient will deteriorate quickly and worsen over several minutes during the simulation.*
     4. *The second simulation will be focused on air entrainment in the ECMO circuit. Air will be injected into the circuit and cause the circuit to stop providing mechanical support to the patient. This will ideally be recognized immediately and communicated to the team. They must quickly take the appropriate steps to correct the problem which include the correct order of clamping the circuit (again taking the patient off ECMO support) and appropriately de-airing the circuit before re-initiating ECMO. Again, this should be done expeditiously, as the patient will deteriorate quickly and again worsen over several minutes during the simulation.*
     5. *The final stages of the scenario involve transport of the patient from the ambulance, into and out of the aircraft, and finally back to the simulated ICU room. Proper use of the timeout and safe patient transfers will be prompted if not utilized to prevent accidental decannulation. Given that one of the overall goals is teaching the transport curriculum; emphasis is again placed on proper use of the provided protocols and checklists for the various transfers. Ideally these are done without prompting and patient transfers go smoothly.*
  2. Anticipated Management Mistakes
     1. **Failure to utilize the transport protocol**
        1. **Facilitator can prompt team throughout the simulation to refer to protocol if not being utilized**
     2. **Failure to augment hemodynamic support prior to planned circuit change**
        1. **Patient will become hypotensive 10 seconds after circuit is clamped**
        2. **Facilitator can say “the patient is getting hypotensive, is there anything you’d like to do?”**
     3. **Failure to utilize timeout during patient transfer**
        1. **Facilitator can prompt** “would you like to assign a team member to watch monitors/cannulas/airway?”
     4. **Failure to augment hemodynamic support during circuit emergencies (pump failure, air entrainment)**
        1. **Patient will become hypotensive 1 min after circuit stops**
        2. **Facilitator can say “the patient is getting hypotensive, is there anything you’d like to do?”**
     5. **Failure to vocalize need to determine source of air entrainment**
        1. **More air will be injected into circuit upon circuit re-initiation**
        2. **Facilitator can prompt “looks like there is more air getting into the circuit, do we know where it is coming from?”**
     6. **Failure to utilize rescue ventilator settings (if team switched patient to ultra-protective low tidal volume settings prior to circuit emergency)**
        1. **Patient will become hypoxemic 1 min after circuit stops**
        2. **Facilitator can say “the patient is getting hypoxemic, is there anything you’d like to do?”**
     7. **Failure to request ambulance to pull over during circuit emergency**
        1. **Facilitator can say “it’s pretty bumpy in here, do you want the ambulance to pull over or keep driving?”**
